# Lot-Size Models with Uncertain Demand Considering Its Skewness/Kurtosis and Stochastic Programming Applied to Hospital Pharmacy with Sensor-Related COVID-19 Data

**DOI:** 10.3390/s21155198

**Published:** 2021-07-31

**Authors:** Fernando Rojas, Víctor Leiva, Mauricio Huerta, Carlos Martin-Barreiro

**Affiliations:** 1 School of Nutrition and Dietetics, Faculty of Pharmacy, Universidad de Valparaíso, Valparaíso 2360102, Chile; fernando.rojas@uv.cl; 2 Center of Micro-Bioinnovation, Faculty of Pharmacy, Universidad de Valparaíso, Valparaíso 2360102, Chile; 3 School of Industrial Engineering, Faculty of Engineering, Pontificia Universidad Católica de Valparaíso, Valparaíso 2362807, Chile; mauricio.huerta@pucv.cl; 4 Faculty of Natural Sciences and Mathematics, Universidad Politécnica ESPOL, Guayaquil 090902, Ecuador; cmmartin@espol.edu.ec or; 5 Facultad de Ingeniería, Universidad Espíritu Santo, Samborondón 0901952, Ecuador

**Keywords:** GAMLSS methodology, machine learning, non-Gaussianity, SARS-Cov2, sensing and data extraction, statistical moments, two-stage optimization algorithms

## Abstract

Governments have been challenged to provide timely medical care to face the COVID-19 pandemic. Under this pandemic, the demand for pharmaceutical products has changed significantly. Some of these products are in high demand, while, for others, their demand falls sharply. These changes in the random demand patterns are connected with changes in the skewness (asymmetry) and kurtosis of their data distribution. Such changes are critical to determining optimal lots and inventory costs. The lot-size model helps to make decisions based on probabilistic demand when calculating the optimal costs of supply using two-stage stochastic programming. The objective of this study is to evaluate how the skewness and kurtosis of the distribution of demand data, collected through sensors, affect the modeling of inventories of hospital pharmacy products helpful to treat COVID-19. The use of stochastic programming allows us to obtain results under demand uncertainty that are closer to reality. We carry out a simulation study to evaluate the performance of our methodology under different demand scenarios with diverse degrees of skewness and kurtosis. A case study in the field of hospital pharmacy with sensor-related COVID-19 data is also provided. An algorithm that permits us to use sensors when submitting requests for supplying pharmaceutical products in the hospital treatment of COVID-19 is designed. We show that the coefficients of skewness and kurtosis impact the total costs of inventory that involve order, purchase, holding, and shortage. We conclude that the asymmetry and kurtosis of the demand statistical distribution do not seem to affect the first-stage lot-size decisions. However, demand patterns with high positive skewness are related to significant increases in expected inventories on hand and shortage, increasing the costs of second-stage decisions. Thus, demand distributions that are highly asymmetrical to the right and leptokurtic favor high total costs in probabilistic lot-size systems.

## 1. Symbology, Introduction, Bibliographical Review, and Objectives

In this section, abbreviations, acronyms, notations, and symbols used in our work are defined in Table 1. In addition, we provide here the introduction, the bibliographical review on the topic about related works, and our objectives, together with the description of the sections considered in this paper.

### 1.1. Abbreviations, Acronyms, Notations, and Symbols

Next, Table 1 presents the acronyms and statistical symbology considered in this paper to facilitate its reading. For more notations related to the stochastic programming (SP) formulation, see Table 2.

### 1.2. Introduction

The Coronavirus disease 2019 (COVID-19) is a severe acute respiratory syndrome Coronavirus 2 (SARS-CoV-2) detected in China in December 2019. COVID-19 arrived in South America in February 2020 [1] and was declared a pandemic by the World Health Organization (WHO) in March 2020 (https://www.who.int/news/item/07-03-2020-who-statement-on-cases-of-covid-19-surpassing-100-000; accessed on 16 July 2021). The COVID-19 pandemic has changed the usual people behavior around the world, and its impact on health and the worldwide economy and finance is notorious [2,3,4]. The number of people affected by COVID-19, the number of lives lost, and the results of the measures taken by government authorities throughout the world are aspects to be studied [5].

The healthcare industry plays an essential role in modern societies [6]. As in most industries, this sector competes in terms of time and quality. However, due to the COVID-19 pandemic, several countries have made efforts to manage the goods and services necessary to control the pandemic. The healthcare industry has played a relevant role facing COVID-19. The time and quality in the administration of this industry can be favored by an efficient inventory management, which gives structure and direction to the decision-making in an organization regarding the supply chain [7]. Around the world, even more in the COVID-19 pandemic, efforts have been made to increase the installed capacity of diagnostics [8], beds for the care of critical patients, and the inclusion of new hospital equipment and supplies, considering drug management. Note that the studies on efficient inventory management in the healthcare industry not only focus on an internal analysis, but an attempt has also been made to investigate this problem from the perspective of supply chain management [9,10,11].

One of the most significant aspects of the supply chain refers to inventory management [12]. In such a management, the costs of purchase, order, holding, and shortages are determined by estimating the demand in a specific time under certain constraints. Such constraints may be of different types: budgetary, storage volume, and service levels, among others [12]. Inventory models are needed to manage a logistically efficient organization, and their objective is to minimize the total cost (TC) as a function of the mentioned costs, stating an economic order quantity (EOQ) or lot size to satisfy demand [13].

When optimization problems involve uncertainty or randomness, SP [14] can be considered. While deterministic optimization problems are formulated with known parameters, real-world problems almost surely include unknown parameters when decisions must be made. Note that, if the parameters are unknown, but they are assumed to be within a set of possible values, a solution may be feasible for all possible values of parameters by optimizing an objective function [15].

Although the lot-size model considers a deterministic demand, it is possible to extend this model to a probabilistic (stochastic) environment through an approach based on two-stage SP [16]. In its first stage, decisions are made without knowing the realization of a random variable, which in our case is the demand per unit of time (DPUT). Subsequently, in a second stage, stock decisions and possible shortages are made, generating stochastic scenarios for the demand of a product [12]. We assume that, at any stage, the number of states of the system is finite and that these states are perfectly described by the corresponding variables (often multidimensional) [17]. However, in general, supply forecasting in hospital pharmacies [18] is done for a short period, so it is better to consider SP models in two stages rather than multiple.

To the best of our knowledge, the probabilistic lot-size models have been mainly studied assuming DPUT of the products which are independent and identically distributed (IID) under a normal/Gaussian model. However, the DPUTs are random variables that may show other shapes that normality/Gaussianity [19]. For the statistical distributions of IID random variables, such as the DPUTs, a statistical moment is a particular calculable dimension of the shape of their probability density function (PDF). The zero-th moment is always equal to one, the first moment is the mean, the second central moment is the variance, the third standardized central moment is the coefficient of skewness or asymmetry (CS), and the fourth standardized central moment is the coefficient of kurtosis (CK) [20,21,22]. As we will see in the background of this paper, all these statistical moments are related mathematically among them. Here, we postulate that such statistical moments can affect the lot size of a probabilistic inventory model.

In the current COVID-19 contingency scenario, drug demand patterns have undergone major changes. The corresponding demand data are often collected through sensors. The changes in drug demand patterns are materialized in two possible cases: (i) some products suffer large increases in the demanded quantities (demand shock), and (ii) others suffer an abrupt drop in these quantities requested for the treatment of patients. Such changes in demand patterns can show an increase in kurtosis and skewness to the right in the statistical distribution of the DPUT data, in the first case, and a decrease in its kurtosis and skewness to the left in the second case. In both cases, such patterns are critical to determine the optimal lots to order and the optimal inventory TCs.

### 1.3. Objectives and Description of the Paper

In this study, we consider the effect of the asymmetry and kurtosis of the DPUT distribution in the availability of critical pharmaceutical products in public hospitals that face COVID-19 contingency issues. The statistical analysis is based on generalized additive models for location, scale, and shape (GAMLSS), which are semi-parametric regression and machine learning models introduced in [23], that allow great versatility in the data description [24]. Particularly, we model the DPUT with a Weibull distribution in a GAMLSS environment, which allows us to fit demand scenarios with different degrees of asymmetry and kurtosis.

Our main contribution in this investigation is to evaluate the effect of asymmetry and kurtosis of DPUT distributions for pharmaceutical products, used during the COVID-19 pandemic, on the inventory TC and decisions of a supply system managed under a stochastic lot-size policy. The gap in the literature, that our study is filling, is how to overcome the intractability of kurtosis and skewness in classical probabilistic inventory models. We use two-stage SP and design a framework for assessing how the shape of the DPUT affects the decision-making. In particular, we affirm that the contexts of therapeutic uncertainty faced by the health system influence advanced care to support patient management [25]. This can be improved with a best management of the useful drugs supply to support COVID-19 therapies, considering an adequate modeling of the demand for pharmaceutical products. Then, the implications of our study are important when making logistics decisions by assuming different DPUT asymmetry and kurtosis patterns in a probabilistic lot-size model of the inventory upon study. Therefore, the objectives of this investigation are: (i) to analyze the changes in the TC and inventory indicators of a probabilistic lot-size model under different DPUT scenarios; and (ii) verify whether the relationships between inventory decisions and CS/CK, obtained via simulation, are confirmed in real-world scenarios.

The rest of this paper is organized as follows. In Section 2, we propose an algorithm that uses sensors to submit requests when supplying products for the treatment of COVID-19 in hospitals. Section 3 presents the methodology proposed in this study built upon two pillars: (i) DPUT distributions with different levels of skewness and kurtosis; and (ii) a probabilistic lot-size model in two stages. A simulation study is performed in Section 4 to assess how the DPUT distribution with different levels of skewness and kurtosis affect the TC and inventory decisions of a probabilistic lot-size model. Section 5 and Section 6 finish our investigation with a discussion and conclusions of the obtained results, along with their limitations and possible future research.

## 2. Pharmaceutical Inventory Management through Sensors for COVID-19

The use of sensors is essential in inventory systems because they allow us, in a planned and automated way, to detect low levels of stock in products stored in the warehouses. Such a sensorization enables us to react quickly to a possible shortage. In hospitals that care for patients with COVID-19, the behavior of the demand for pharmaceutical products must be studied to provide timely supply and adequate care. We propose to use sensors for the replenishment of this type of critical products.

The stock of a particular product varies according to its demand. This is why it is important to program sensors in the repository where the data of the different products are stored and so acting in the event of a possible shortage.

In a database, stock movements of the products are stored as records in a table. To incorporate a sensor associated with a product table, a trigger can be programmed. Note that a trigger is a piece of code that reacts when any change is made to a table, for example, when the availability of a product in inventory changes. In particular, in the case of a hospital that works with pharmaceutical products for patients with COVID-19, each time a product is withdrawn from inventory, its future availability must be sensed. When this stock is below a threshold (preset by product), the trigger warns by sending a signal that indicates that there is a risk of shortage for a product and also that a supply order must be made. The trigger is an important component of the sensor and is stored together with the data in the database.

**Figure 1:**
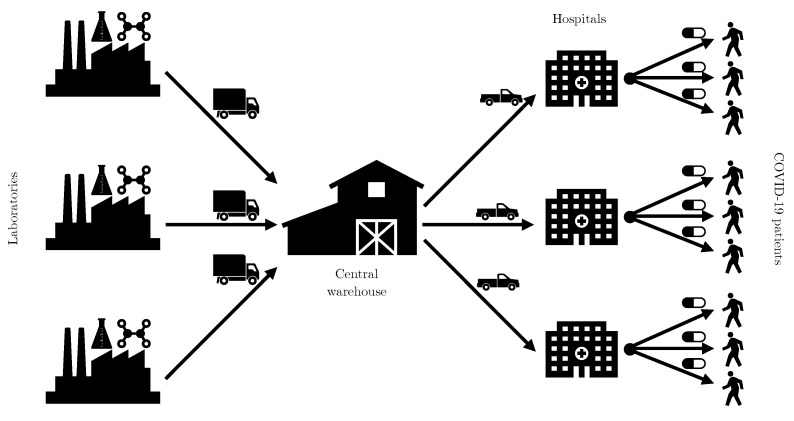
Scheme of drug demand data collected through sensors.

Algorithm 1 introduces a method that uses sensors in the hospital’s databases and in the database of a central warehouse (see Figure 1) to replenish the inventories of the hospitals that treat patients with COVID-19. The demand data for COVID-19 drugs are often collected through sensors in a setting similar to the scheme provided in Figure 1.
**Algorithm 1:** Approach for replenishment of pharmaceutical products for COVID-19 in hospitals using sensors.1:Update for each hospital, and for each new inventory period, the initial stock of the products taking the final stock of the previous period.2:Generate for each hospital, and for each product delivered to a patient, the corresponding withdrawal in the hospital database, that is, the available stock of the product is decreased by the number of units in which it has been delivered.3:Monitor by a sensor in the hospital’s database the current stock for every product delivered to a patient. If the stock of a particular product is below the threshold:
3.1:The trigger generates a product supply order. This order is received by a central warehouse whose role is to supply the hospitals.3.2:From the central warehouse, the orders from the different hospitals are accumulated in a principal database that has a sensor. The trigger of this sensor generates the purchase orders to the different suppliers when each product reaches its threshold in this global repository.3.3:The central warehouse receives the products from the suppliers and sends the products to the hospitals.3.4:The stock of each received product is updated for each hospital.

## 3. Methodology

In this section, the proposed methodology is divided as follows:

- Section 3.1, A GAMLSS Formulation: This subsection introduces the GAMLSS formulation for modeling any parameter of a DPUT distribution.
- Section 3.2, Moments, Skewness, and Kurtosis: This subsection defines the statistical moments and CS/CK, showing how to obtain them for the Weibull type 3 distribution.
- Section 3.3, The Probabilistic Lot-Size Model: This subsection provides the elements of a probabilistic lot-size inventory model using SP in two stages.

### 3.1. A GAMLSS Formulation

The GAMLSS are a modern family of statistical and machine learning models based on the distribution of the response variable focused on the semi-parametric regression analysis. Such as in the generalized linear models (GLM), in the GAMLSS, a discrete or continuous parametric distribution can be assumed for the response variable, but the parameters of this distribution may vary according to the covariates using linear, non-linear, or smoothed functions. This allows the GAMLSS to learn more general patterns in the relationships between covariates and the response variable in contrast to the GLM, improving the prediction of the response. For an overview of limitations and features of the GLM in contrast to the GAMLSS, see the book [26].

In the GAMLSS framework, the exponential-family assumption for the response variable, essential in the GLM, is relaxed and replaced by a general distribution family. Instead, a wide range of distributions can be considered, including probabilistic models with heavy or light tails. This permits us to model a wide range of levels of asymmetry and kurtosis. In addition, all parameters of the distribution, such as location (for example, the mean), scale (for example, the variance), and shape (for example, the skewness and kurtosis), may be modeled as linear, non-linear or smoothed functions of the covariates [27]. This possibility of modeling shape parameters enables us to study the skewness and kurtosis parameters in our work. Furthermore, the GAMLSS not only support continuous and discrete distributions, but also mixture and zero/one inflated distributions can be modeled, including overdispersion. This opens new avenues that may be of interest for future research. Then, the GAMLSS are especially suited for describing a leptokurtic/platykurtic and positive/negative asymmetric distributions of a response variable, for example, the DPUT with data obtained from sensors. The current packages available in the R software [28] allow us to work with distributions belonging to the GAMLSS family. The models can be selected according to goodness-of-fit criteria when fitting real-world data, as well as by generating random numbers with arbitrary distributions of interest for theoretical or empirical research [12,29,30].

We are interested in modeling the DPUT using a GAMLSS framework due to its flexibility and the possibility of modeling skewness and kurtosis. Specifically, let *Y* be a random variable corresponding to the DPUT of an inventory product. Then, consider that μ=E[Y] is its expected value, given a set $D_{1}$ of covariates.

Let f(y;θ) be the PDF of the DPUT *Y* with parameter θ, and F(y;θ) be the corresponding cumulative distribution function (CDF), where $\theta ={\left(\mu ,\sigma ,\nu ,\tau \right)}^{⊤}={\left(\theta_{1},\theta_{2},\theta_{3},\theta_{4}\right)}^{⊤}$ is a vector of four distribution parameters. The first two parameters μ and σ usually characterize the location and scale, while ν and τ are shape parameters (for example, associated with skewness and kurtosis). Thus, a GAMLSS structure for every statistical parameter is formulated as

$$\begin{array}{ccc} g_{1}\left(\mu \right) & = & \eta_{1}=D_{1}\beta_{1}+\sum_{j=1}^{J_{1}}h_{j1}\left(d_{j1}\right),\\ g_{2}\left(\sigma \right) & = & \eta_{2}=D_{2}\beta_{2}+\sum_{j=1}^{J_{2}}h_{j2}\left(d_{j2}\right),\\ g_{3}\left(\nu \right) & = & \eta_{3}=D_{3}\beta_{3}+\sum_{j=1}^{J_{3}}h_{j3}\left(d_{j3}\right),\\ g_{4}\left(\tau \right) & = & \eta_{4}=D_{4}\beta_{4}+\sum_{j=1}^{J_{4}}h_{j4}\left(d_{j4}\right), \end{array}$$

where μ,σ,ν,τ, and $\eta_{k}$ are n×1 vectors of the parameters of interest conditioned to an $n\times J_{k}$ known matrix of covariates $D_{k}=\left(d_{1k},\ldots ,d_{J_{k}k}\right)$, for k=1,2,3,4, whereas $\beta_{k}$ are $J_{k}\times 1$ vectors of the regression coefficients to be estimated. In addition, $g_{k}$ is the *k*-th link function used to model the respective parameter, and $h_{jk}$ are semi-parametric additive functions for the covariate $d_{jk}$. For mathematical details of the parameter estimation, diagnostics, and goodness of fit in a GAMLSS setting, see [23].

### 3.2. Moments, Skewness, and Kurtosis

The statistical moments of a random variable *Y* with CDF *F* are the expected values of a function on *Y*. These moments represent statistical measures that characterize a random variable. Using moments for characterizing a random variable is a helpful procedure, especially if the probability distribution is unlikely to be known. Although the moments can be defined around any point, the most common is calculating them around zero (simply or raw moments) or around the expected value of the random variable (central moments). Considering the Riemann-Stieltjes integral [31], for any integer value *r*, the *r*-th moment of a continuous statistical distribution is defined as

(1) $$\mu^{\left(r\right)}=E\left[Y^{r}\right]=\int_{-\infty }^{\infty }y^{r}\;dF\left(y\right),$$

with E denoting the expectation operator. Usually, the first moment is related to the location of the distribution of the corresponding random variable. Assuming that the moments exist for a distribution, every value of $\mu^{\left(r\right)}$ represents a characteristic of the distribution of the random variable. This is because, given the uniqueness of the corresponding integral stated in (1) (or summation in the discrete case), if the *r*-th moment of a random variable exists, then it is unique. Thus, every distribution has a unique succession of moments, and vice versa. From the expression defined in (1), it is easy to observe that the zero-th moment of any distribution is equal to one, and then the first raw moment is the mean μ=E[Y].

If E[Y] exists, then we can define its *r*-th central moment as the expected value of the difference between the random variable *Y* and its mean μ, raised to the *r*-th power, that is,

(2) $$\mu^{\prime (r)}=E\left[{\left(Y-\mu \right)}^{r}\right]=\int_{-\infty }^{\infty }{\left(y-\mu \right)}^{r}\;dF\left(y\right).$$

From (2), note that the second central moment is the variance of *Y*, $\mathrm{Var}\left[Y\right]=E[{\left(Y-\mu \right)}^{2}]$ namely. Then, the second central moment can be interpreted as a scale or dispersion metric of a random variable. It is known that the square root of the variance is the standard deviation (SD), that is, $\mathrm{SD}\left[Y\right]={\left(E\left[{\left(Y-\mu \right)}^{2}\right]\right)}^{1/2}.$ In particular, the SD is used to compute the *r*-th standardized central moment $\mu^{\prime \prime (r)}$ of the random variable *Y*, defined as

$$\mu^{\prime \prime (r)}=\frac{\mu^{\prime (r)}}{{\left(\mathrm{SD}\left[Y\right]\right)}^{r}}=\frac{E\left[{\left(Y-\mu \right)}^{r}\right]}{{\left(\mathrm{SD}\left[Y\right]\right)}^{r}},\;r=1,2,\cdots ,$$

which can also be used for characterizing the distribution of *Y*. Note that the standardized central moments are dimensionless (free from measurement units).

The third standardized central moment of *Y* is its CS, that is, $\mathrm{CS}\left[Y\right]=\mu^{\prime \prime (3)}$. If CS[Y] takes a negative value, then the distribution of *Y* has a left tail larger than the right tail and is said to have a negative asymmetry or to the left. Similarly, if CS[Y] takes a positive value, then the distribution of *Y* has a right tail larger than the left tail and is said to have a positive asymmetry or to the right. If CS[Y]=0, then the distribution of *Y* is symmetrical around the mean μ.

The fourth central moment is a measure to identify outliers which are far from the mean μ. Typically, this measure is represented by the fourth standardized central moment, also called the Fisher CK of *Y*, denoted as $\mathrm{CK}\left[Y\right]=\mu^{\prime \prime (4)}$. Statistical distributions with a CK[Y]<3 are said to be platykurtic having a more flattened shape than the standard normal distribution. By contrast, distributions with a CK[Y]>3 are said to be leptokurtic with a pointed shaped different from the standard normal distribution.

Note that the only case when CS[Y]=0 and CK[Y]=3 corresponds to the normal distribution. In addition, recall that CS and CK can be interpreted as shape measures of the statistical distribution.

### 3.3. The Probabilistic Lot-Size Model

To obtain the observed values of a forecasted DPUT $y_{t}^{\left(\omega \right)}$ of $Y_{t}$, their probabilities $p_{t}^{\left(\omega \right)}$, and E(TC), we adapt the method proposed in [12] to a GAMLSS environment. The corresponding SP framework used to minimize E(TC) of the inventory model can be formulated as [15]

(3) $$\min\left\{E\left(\mathrm{TC}\right)\right\}=\min\left\{\sum_{\omega \in \Omega }\sum_{t=T}^{T+1}o_{t}Z_{t}+u_{t}Q_{t}+p_{t}^{\left(\omega \right)}\left(h_{t}I_{t}^{\left(\omega \right)}+s_{t}S_{t}^{\left(\omega \right)}\right)\right\},$$

subject to

(4) $$\begin{array}{ccc} Q_{t}+\left(I_{t-1}^{\left(\omega \right)}-S_{t-1}^{\left(\omega \right)}\right)-\left(I_{t}^{\left(\omega \right)}-S_{t}^{\left(\omega \right)}\right) & = & y_{t}^{\left(\omega \right)},\\ u_{t}Q_{t} & \leq & C_{t}Z_{t}, \end{array}$$

$$\forall t\in T,\;\;\omega \in \Omega ,\;\;Q_{t}\geq 0,\;\;I_{t}^{\left(\omega \right)}\geq 0,\;\;S_{t}^{\left(\omega \right)}\geq 0,\;y_{t}^{\left(\omega \right)}\geq 0,\;\;p_{t}^{\left(\omega \right)}\in \left[0,\;1\right],\;\;Z_{t}\in \left\{0,\;1\right\},$$

where Ω corresponds to the set of probable scenarios ω of the DPUT, with a given number of scenarios in each period of the decision stages. Table 2 summarizes the elements of the SP model. In this way, it is possible to minimize E(TC) in all scenarios and periods. To optimize the objective function stated in (3) under the L-shaped method, we need to add feasibility and optimality cuts to this function and its constraints [32].

## 4. Simulation Study and Real Pharmaceutical Case Study

The new methodology proposed in this paper is studied using simulation and corroborated by a case study applied to the pharmaceutical industry. Here, we consider the following three subsections:

- Section 4.1, Computational Framework and Simulation Scenarios: This subsection presents details of the used computational framework and describes the simulation scenarios, which is divided into two parts, as indicated below.
- Section 4.2, Results of the Simulation Study: This subsection provides the results of simulation study where we analyze the performance of a new methodology of probabilistic lot-size models on inventory with different levels of skewness and kurtosis for the demand distribution.
- Section 4.3, Pharmaceutical Case Study in a COVID-19 Scenario: This subsection reports an analysis of a case study with real-world data.

### 4.1. Computational Framework and Simulation Scenarios

To evaluate our proposal, we simulate 10,000 scenarios to establish different: (i) inventory parameters; (ii) statistical models for the DPUT; and (iii) SP formulations to minimize costs. The computational routines were implemented in a non-commercial software named R in its version 4.0.3. For more details of this software, see http://www.r-project.org (accessed on 16 July 2021), and, for R packages related to inventory models, see [30,33,34,35,36]. The computer specifications used in these experimental results are reported in Table 3. For example, in a solution where 100 DPUT scenarios are occupied in a single decision period, the runtime was of 10 s.

In this study, we use a probabilistic lot-size model adapted from [12]. The associated statistical modeling is based on IID DPUTs assuming a Weibull type 3 (WEI3) statistical distribution of parameters μ and σ, with PDF stated as

$$f\left(y;\mu ,\sigma \right)=\left(\frac{\sigma }{\beta }\right){\left(\frac{y}{\beta }\right)}^{\sigma -1}\exp\left(-{\left(\frac{y}{\beta }\right)}^{\sigma }\right),\;y>0,\mu >0,\sigma >0,$$

where β=μ/(Γ(1/σ)+1). In addition, we can calculate the variance of the WEI3 distribution as

(5) $$\mathrm{Var}\left[Y\right]={\left(\mathrm{SD}\left[Y\right]\right)}^{2}=\mu^{2}\left(\frac{\Gamma (\frac{2}{\sigma +1})}{\Gamma {\left(\frac{1}{\sigma +1}\right)}^{2}}-1\right),$$

with Γ denoting the usual gamma function. Note that, if we get a random sample of size *n*, namely $Y_{1},\ldots ,Y_{n}$, from a WEI3 distribution, and consider a known design matrix $D_{k}$ of $J_{k}$ covariates, then the statistical parameters can be estimated by using the model described in Section 3.1[37]. Without loss of generality, neither covariates nor additive functions are considered in the simulation study. This implies that μ and σ are no conditioned, and they are unique for all elements of the random sample $Y_{1},\ldots ,Y_{n}$. Thus, we denote these parameters with the usual notation for scalars from now on, that is, μ and σ, respectively.

In this work, we do not use the popular and widely known normal distribution. Instead, we employ the WEI3 distribution since this gives us the possibility of generating asymmetric scenarios and with different degrees of kurtosis. Hence, we assume several levels of skewness and kurtosis with the WEI3 distribution for the DPUT. The uniformly distributed parameters employed to build these scenarios are chosen from values found in selected papers; see [38] for a list of these values. Once the inventory and statistical models are stated, we utilize SP to minimize the TC of inventory by using the objective function formulated in Section 3.3.

Table 4 details the statistical and inventory parameters to generate 10,000 scenarios for inventory policies obtained by SP in two stages. The choice of the values in this table is based on previous studies on the topic [38,39,40]. Algorithm 2 summarizes the approach considered to obtain the TC over one period of the decision stages using SP in two stages. We perform a cluster analysis of DPUT scenarios using the RcmdrMisc package and optimize the objective function stated in (3) with the lpSolveAPI package.

### 4.2. Results of the Simulation Study

Firstly, we analyze 10,000 scenarios with different levels of skewness and kurtosis for the DPUT distribution with respect to the probabilistic lot-size model that provide the minimum TC, and the decision in first and second stages. Table 5 reports the descriptive statistics for the indicators of the 10,000 scenarios of the simulation study. Subsequently, the correlation between variables of the simulation study was evaluated using the Pearson correlation coefficient matrix. These correlations are shown in Table 6. An extract of correlations of interest for this study according to their significance given by the corresponding *p*-value is reported in Table 7.    
**Algorithm 2:** Summary of the methodology to optimize TC over one period of decision stages, using SP in two stages1:Collect data $y_{1},\ldots ,y_{n}$ of the random variable *Y* under study, in our case the DPUT.2:Fit the WEI3 distribution to the data collected in step 1 according to the following steps:2.1Compute descriptive statistics for $y_{1},\ldots ,y_{n}$ by using the histDist command of the gamlss package.2.2Consider a suitable link function for the GAMLSS based on step 2.1.2.3Estimate the GAMLSS parameters (θ) using the maximum likelihood method.2.4Obtain the 50-th percentile, namely $y_{50}$, of the distribution of *Y* using the estimates of the parameter θ calculated in step 2.3.3:Generate cluster scenarios carrying out a “for cycle” with t=1 period of the decision stages following the steps:3.1Fix $l=l_{1}$ at a median value ($l_{1}\times 100=50$) and simulate *n* data $y_{1_{l_{1}\times 100}}^{⋆},\cdots ,y_{n_{l_{1}\times 100}}^{⋆}$ of the distribution of *Y* using the Monte Carlo method, with estimated parameters computed in step 2.3.3.2Conduct a cluster analysis on $y_{1_{l{}_{1}\times 100}}^{⋆},\ldots ,y_{n_{l_{1}\times 100}}^{⋆}$ with a number of 100 scenarios conformed for the clusters and obtain $y_{t}^{\left(\omega \right)}$ and $p_{t}^{\left(\omega \right)}$, for $\omega =1,\ldots ,2^{t}$, where $y_{t}^{\left(\omega \right)}$ is the ω-th cluster centroid (scenario), and $p_{t}^{\left(\omega \right)}$ is the probability of occurrence of this scenario.4:Set values for the components $u_{t}$, $o_{t}$, $h_{t}$ and $s_{t}$ of the inventory model given in (3) and the components $C_{t}$ and $I_{0}$ of the constrains stated in (4).5:Optimize the model formulated in (3) to obtain $Z_{t}$, $Q_{t}$, $I_{t}^{\left(\omega \right)}$ and $S_{t}^{\left(\omega \right)}$, denoted by ${\tilde{Z}}_{t}$, ${\tilde{Q}}_{t}$, ${\tilde{I}}_{t}^{\left(\omega \right)}$ and ${\tilde{S}}_{t}^{\left(\omega \right)}$, respectively.6:Establish the optimum inventory TC as
$\tilde{E}\left(\mathrm{TC}\right)=\sum_{\omega \in \Omega }^{}\sum_{t=1}^{T}p_{t}^{\left(\omega \right)}\left(o_{t}{\tilde{z}}_{t}+u_{t}{\tilde{Q}}_{t}+h_{t}{\tilde{I}}_{t}^{\left(\omega \right)}+s_{t}{\tilde{S}}_{t}^{\left(\omega \right)}\right).$7:Report the results obtained.

Table 8 shows the results of linear regressions between variables with significant correlations. Note that the inventory stock (*I*) decreases as CK[Y] increases in positive values. In addition, the mean of DPUT (μ) increases as *I* increases. Observe that CS[Y] is directly related to *I*, so that large values of CS[Y] tend to take high positive values of *I*. In addition, as CK[Y] takes large negative values, it generates high shortage (*S*). When *I* increases, μ increases, and *S* increases, as well. This relationship is important to be considered since, according to the adjusted R-squared value shown in Table 8, a 29.82% of the shortage is explained by μ. Therefore, positive values of CS[Y] generate shortages. However, negative values of CS[Y] result in negative values of shortage, which is interpreted as over-balance stock.

**Figure 2:**
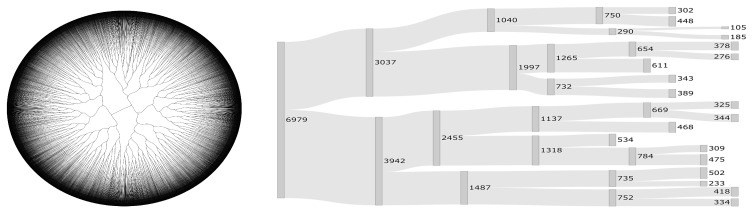
Visualization of the cluster analysis of the variables in the simulations through circular dendrogram (**left**) and Sankey dendrogram (**right**), with the thickness of the band proportionally representing the size of each cluster in each split procedure.

To form groups of scenarios that are homogeneous among themselves, but heterogeneous compared to the rest of groups, a hierarchical cluster analysis was carried out by using similarity of multiple variables. This cluster is illustrated in the dendrogram presented in Figure 2. This dendrogram allows us to visualize the variables on which the scenarios are formed in four groups. Due to the high dimensionality of the data, the dendrogram of Figure 2 (left) was made using a circular representation through polar coordinates, which allowed us to show the full tree. However, when the sample is large, it is still difficult to observe the behavior of the clustering process. To improve the visualization of our dendrogram, Figure 2 (right) shows a pruned version of it using a Sankey plot. To the best of our knowledge, no Sankey plot has been used before to represent a high-dimensional dendrogram for clustering. We call this plot as Sankey dendrogram. In this figure, the thickness of the band proportionally represents the size of each cluster in each split procedure; see values in this figure. We represent each split through the boxes, including the value of the respective cluster size.

Subsequently, a cluster analysis called k-means is carried out, which quantified the number of scenarios by clusters, in addition to averaging their variables called centroids, thus facilitating the data analysis. To confirm this, it is necessary to study the variability of the groups given by the sum of squares, resulting in an intra-group variability of $9.95\times 10^{17}$ and an intergroup variability of $1.10\times 10^{19}$. The intergroup variability is greater than the intra-group variability, which indicates to us that there are effectively four different groups of data. To support our good behavior of the number of clusters, we also compute the silhouette measure, defined as

$$\mathrm{Sil}=\frac{1}{n}\sum_{i=1}^{n}\frac{a_{i}-b_{i}}{\max\{a_{i},b_{i}\}},$$

where $a_{i}$ and $b_{i}$ are the distances between the *i*-th datapoint with its closest and second closest centroids, respectively. High values of silhouette show a good performance of the cluster. In our case, we obtain a silhouette value of 0.64, which can be interpreted as a good performance. The centroids of the generated clusters of scenarios are displayed in Table 9. Note that an important finding was detected when comparing the averages of the variables by the cluster analysis. Group 4 has the highest TC, with the most negative value of the CS and the smallest value of the CK. In addition, this group has the largest shortage and inventory stock and is the group with the highest demand and quantity to buy. In addition, Group 2 has the smallest TC, the largest value of the CK, the smallest shortage, and the largest value of the CS. Therefore, the probability that a scenario belongs to one of the groups is determined using a multinomial logistic regression model, whose values of the regression coefficients are reported in Table 10.

As a way to better interpret the multinomial logistic regression for the probability of belonging to groups 2 and 4, which presented minimum and maximum TCs, respectively, we show an evaluation of these probabilities for minimum and maximum values of CS and CK. This is shown in Table 11. Given the characteristics of the high TC, inventory stock and shortage of group 4, the probability of belonging to this group increases as the CK increases. However, there is no significant increase in probability when taking a high CS. Likewise, only the CK determines an increase significantly in group 2 membership when the CK takes higher values.

### 4.3. Pharmaceutical Case Study in a COVID-19 Scenario

In this case study, we perform a statistical analysis of a DPUT data set for a pharmaceutical product named “Salbutamol”, which is an inhaler used as palliative COVID-19 treatment in an anonymous Chilean public hospital pharmacy. The data set is available from the authors under request. We optimize an inventory system for this pharmaceutical service and reduce costs associated with the decisions based on the probabilistic lot-size model. The DPUT data sample corresponding to monthly demand of Salbutamol inhalers was obtained by means of a non-random procedure called convenience sampling. The sample was collected between January and December of the years 2018–2020 to show the changes of DPUT between the pre-pandemic and pandemic periods, occupying the sensor’s system of a central data warehouse, as indicated in Algorithm 1. The DPUT data set has a size of 36 months, and its descriptive statistics are reported in Table 12.

Note that the empirical DPUT distribution has a leptokurtic shape with a negative skewness. If we assume a WEI3 distribution for modeling the DPUT, the distribution parameters can be estimated by using the histDist command of the gamlss package. In addition, we can calculate the variance using the expression defined in (5); see results in Table 13.

**Figure 3:**
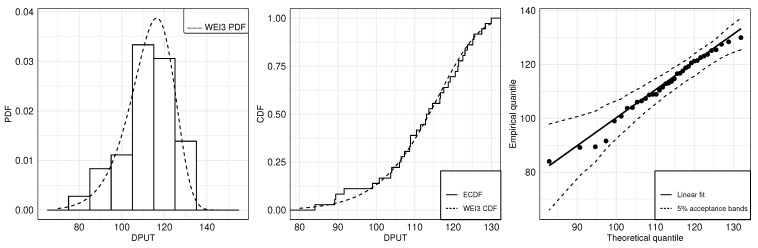
Histogram (**left**) and ECDF (**center**) with the respective fitted WEI3 PDF and CDF; and QQ plot (**right**) for the WEI3 distribution with monthly drug DPUT data.

Figure 3 shows the histogram of the data with a fitted theoretical WEI3 PDF (left), the empirical CDF (ECDF) with the fitted theoretical WEI CDF (center), and the empirical quantile versus theoretical quantile (QQ) plot (right) to check the good fit of the WEI3 distribution to the DPUT data. The QQ plot allows us to graphically evaluate the performance of a proposed probability model adjusted to the data. Since, in the QQ plot, all values are within the acceptance bands, we conclude that the proposed statistical distribution fits the data adequately. Note that no DPUT values seem to behave as outliers of the proposed statistical distribution in the QQ plot, which serves to illustrate the behavior of skewness and kurtosis in this case. Furthermore, the good fit of the WEI3 distribution to the DPUT data also is visualized into the histogram and CDF plot. The coefficients required for carrying out the two-stage SP formulation in a single period are reported in Table 14 with the results of TC, $Z_{t}$, $Q_{t}$, $S_{t}$, and $I_{t}$.

Note that, if we compare the results of our probabilistic lot-size model and a simple EOQ model with shortage, where

$$\mathrm{TC}=\frac{o_{t}\mu }{Q_{t}}+\frac{h_{t}{\left(Q_{t}-S_{t}\right)}^{2}}{2Q_{t}}+\frac{s_{t}S_{t}^{2}}{2Q_{t}}+u_{t}Q_{t}=\$13,440,624.00,$$

with

$$Q_{t}=\sqrt{\frac{2o_{t}\mu }{h_{t}}\left(h_{t}+s_{t}/s_{t}\right)}=3509.86\mathrm{units},$$

and

$$S_{t}=\sqrt{\frac{2o_{t}\mu }{s_{t}}\left(h_{t}/s_{t}+h_{t}\right)}=0.85\mathrm{units},$$

we can conclude that the two-stage SP formulation leads to a smaller TC for the demand data of our pharmaceutical case study, with a smaller lot-sizing, as well. In addition, with this case study, we support the findings of the simulation study, where a large negative value of the CS and a small CK are related to higher shortages and inventory stocks, which are not as high as those calculated by the EOQ model with shortages.

To evaluate the performance of the purchase plan, depending on the lot size obtained by applying the two-stage SP, it is possible to compare this optimal solution with those obtained in a realistic framework of the moving horizon using out-of-sample scenarios. This is done by splitting the sample of DPUTs into a training fraction to estimate the parameters of the theoretical statistical distribution, obtaining the stochastic solution and comparing it with a deterministic real-scenario solution of the remaining fraction of the out-of-sample DPUT. Thus, the percentage increase in the TC that we pay for ignoring the uncertainty is obtained [41].

Similarly, we can use a robust optimization approach employed in problems under uncertainty, where the modeler aims to make decisions that are optimal for the realization in the worst case of the uncertainties within a given set. Usually, the original uncertain optimization problem becomes an equivalent deterministic form (called a robust counterpart) employing strong duality arguments and can then be solved using the optimization algorithms already outlined in this work [42]. Under this context, we can calculate the difference of costs between deterministic and stochastic solutions in relation to the stochastic reference solution defined as

$$\Delta =\frac{\left(\mathrm{DC}-\mathrm{SC}\right)}{\mathrm{SC}},$$

where DC and SC are the TCs obtained for the deterministic and stochastic solutions, respectively.

If we consider *J* repeated instants for this calculation, we can obtain the expected percentage increase in the cost of the deterministic and stochastic solutions, namely E(Δ), and their mean absolute deviation (MAD) as

$$E\left(\Delta \right)=\frac{1}{J}\sum_{j=1}^{J}\left(\frac{{\mathrm{DC}}_{j}-{\mathrm{SC}}_{j}}{{\mathrm{SC}}_{j}}\right)\times 100\%,\;\mathrm{MAD}=\frac{1}{J}\sum_{j=1}^{J}|\frac{{\mathrm{DC}}_{j}-{\mathrm{SC}}_{j}}{{\mathrm{SC}}_{j}}|\times 100\%.$$

We use 35 out of 36 observations to estimate the parameters of the GAMLSS structures. Then, we make predictions for one period of future decision stages out-of-sample and solve the SP as indicated in Algorithm 2 to compare it with the deterministic and robust solutions. The results of E(Δ) and MAD are reported in Table 15. These results agree with our simulation results, indicating that the SP out-of-sample performance is better than the deterministic solution, and that its robust counterpart.

## 5. Discussion

Under the COVID-19 pandemic, the decision-making plays a vital role when setting up timely medicine availability at a low cost since an inadequate decision could affect an entire country. Hospitals do have a critical role in their ability to rapidly deploy a large number of attentions to patients, supply clinical services in emergencies, and support local clinics and laboratories, as well as decrease the risk of a communicable virus that is transmitted from person-to-person by contact. All these factors should be kept in mind to design and control novel inventory policies for policy-makers and governments to execute better decision-making in times of crises, such as upon the COVID-19 pandemic. This study provided a road-map by using novel stochastic inventory optimization methods for policy-makers to mitigate inventory stock-outs or accumulations of critical supplies, while significantly improving their availability and efficiency.

Particularly in the context of therapeutical uncertainty that has been caused by the COVID-19 pandemic, many drugs have experienced explosive demand, and others abruptly have ceased to be occupied. Then, our main contribution in this study was to evaluate the effect of asymmetry and kurtosis of DPUT distributions for pharmaceutical products used during the COVID-19 pandemic, specifically, by evaluating such an effect on the total cost and inventory decisions of a supply system managed under a probabilistic lot-size policy, improving advanced care to support patient management through a more accurate and cost-optimal drug supply.

The scope of this investigation is open to both the academic world in the area of operations research and the practical management of decisions made in the real world. In comparison with previous works, our results suggest new decision-making patterns, such as those considering the levels of skewness and kurtosis of the data distribution related to DPUT. These different levels have shown their effect on the variance, which interacts with the best lot sizing, total cost and the balance or shortages of the products. And, more precisely, with a predictive pattern of the first and second stage decisions, that is, the expected quantities in stock and shortages for the use of stochastic lot-sizing. Our results demonstrated that the higher total cost of supply and greater shortage are related to demand patterns with more negative symmetry and low kurtosis.

Next, we discuss the implications of our results for decision-making in the field of logistics by first referring to the importance of considering skewness and kurtosis patterns that DPUT data can have when analyzing the inventory lot-size. Based on the obtained results, we conclude that these skewness and kurtosis patterns have a direct influence on parameters related to the variance of the DPUT. A simple way to detect skewness and kurtosis patterns of the DPUT is using graphical plots as histograms. For an interpretation of these graphs, the interested reader is referred to [43]. The main implications of the asymmetry and kurtosis levels are the following:

(i) In general, the asymmetry and kurtosis of the DPUT distribution do not seem to affect first-stage lot-sizing decisions.
(ii) DPUT distributions with negative skewness (CS <0) and leptokurtic, that is, to say more pointed and with thicker tails than the normal distribution (CK >3), favor high total costs in probabilistic lot-size systems. These levels of asymmetry and kurtosis of the DPUT distribution are associated with an increase in the variance of the DPUT random variable.
(iii) Increments of one unit in the CK, that is, more leptokurtic DPUT distributions, have a diffuse effect on the expected stored quantities, which we recall was denoted by *I*, and shortage, denoted by *S*, causing decreases of 12% of *I*, with predictive power of 9%, and a 13% increase in *S*, with a slightly smaller predictive power of 8.69%.
(iv) Increments of one unit in the CS, that is, a DPUT distributions with more positive skewness, have a strong effect on *I* and *S*, causing increases of 67.38% for *I*, with predictive power of 18.9%, and a 68.07% of increase for *S*, with a predictive power of 18.27%, increasing the costs of second-stage decisions.

As the most relevant implications for decision-making about probabilistic lot-size of products, derived from the shape of the DPUT distribution, correspond to second-stage decisions, we recommend the use of higher safety inventory levels that protect from shortages, in the case of scenarios with leptokurtosis and a high asymmetry to the right.

## 6. Conclusions, Limitations, and Future Research

Given the therapeutic uncertainty that the COVID-19 pandemic has caused, it has become necessary to study new ways to model the demand for pharmaceutical products useful in its treatment, and for others that are no longer in demand in these scenarios. In this paper, we have shown relationships between the skewness and kurtosis of the DPUT distributions, with respect to the second stage decisions of a probabilistic lot-size model. Coefficients that define the shape of the DPUT distribution would have no effect on first-stage lot-size decisions. We have designed an algorithm that allows us to use sensors when submitting requests for the supply of pharmaceutical products in the hospital treatment of COVID-19.

The main limitations of our study have been to use a simulation approach based on probabilities of demand scenarios not conditioned to previous events of this variable. We have not explored what could happen if the variability of demand is increased or decreased in the period of time studied. Another limitation is that the simulation model is not able to be used outside the limits of the parameter space by means of which such a model was constructed. This may cause a false appreciation of the probabilistic lot-size in a two-stage problem when occupying other scenarios. Assuming the limitations of this work, which have to do with the non-consideration of heterogeneity of the DPUT and the time-dependent scenarios, our investigation is expandable to more general models with heteroscedasticity of variance, such as the widely known variants of generalized lineal models, which are very flexible allowing linear and non-linear functional structures. In addition, in line with the present investigation, the methodology to model a time series, such as the generalized autoregressive and moving average models, can be explored, as well as effects over lot-size decisions [44]. Furthermore, multivariate time series may be also considered. Some of these issues are being analyzed by the authors, whose findings will be reported in future articles.

Our future research trend will focus more on cost saving, inventory availability, and uninterrupted supply, as well as dealing with unexpected/unpredicted change in the demand. Our study can be regarded as a pioneer in this research perspective by investigating the lot-size decisions through considering how the skewness and kurtosis levels of the data distribution may affect the decision-making in probabilistic lot-size inventory models.

## Figures and Tables

**Table 1:** Abbreviations, acronyms, notations, and symbols employed in the present document.

Abbreviations/Acronyms		Notations/Symbols	
CDF	cumulative distribution function	*Y*	DPUT random variable
CK	coefficient of kurtosis	∼	distributed as
COVID-19	Coronavirus disease 2019	Y∼ WEI3	*Y* is WEI3 distributed
CS	coefficient of skewness	E[TC]	expected total cost
DPUT	demand per unit of time	$\mu^{\left(r\right)}=E\left[Y^{r}\right]$	*r*-th moment of *Y*
ECDF	empirical CDF	μ=E[Y]	mean of *Y*
EOQ	economic order quantity	Var(Y)	variance of *Y*
GAMLSS	generalized additive models for	$\sqrt{\mathrm{Var}[Y]}$	SD of *Y*
	location, scale, and shape	CS[Y]	CS of *Y*
GLM	generalized linear models	CK[Y]	CK of *Y*
IID	independent and identically distributed		
MAD	mean absolute deviation		
PDF	probability density function		
QQ	quantile versus quantile		
SARS-CoV-2	severe acute respiratory syndrome		
	Coronavirus 2		
SD	standard deviation		
TC	total cost		
DC	deterministic total cost		
SC	stochastic total cost		
WEI3	Weibull type 3 distribution		
WHO	World Health Organization		

**Table 2:** Model parameters and decision variables of the lot-size model.

Parameters		Variables	
*t*:	Period index of the decision stage in the	$Z_{t}$:	Binary variable indicating whether
	planning time horizon (t=1,⋯,T).		a purchase is carried out in period *t* or not.
$C_{t}$:	Purchase budget in period *t*.	$Q_{t}$:	Quantity of units to be purchased in period *t*.
$u_{t}$:	Unitary cost of purchase in period *t*.	$I_{t}$:	Stock level at the end of period *t*.
$o_{t}$:	Fixed order cost in period *t*.	$I_{0}$:	Initial stock level.
$h_{t}$:	Holding cost at the end of period *t*.	$S_{t}$:	Shortage level at the end of period *t*.
$s_{t}$:	Shortage cost at the end of period *t*.		
$p_{t}^{\left(\omega \right)}$: Probability of occurrence of the scenario ω in period *t* of the decision stage.			

**Table 3:** Characteristics and its description of the computer used in the experimental studies.

Characteristic	Description
Operating system	Windows 10 home single language 64 bits (10.0, compilation 17134)
Model	80FY, BIOS A7CN44WW
Processor	INTEL (R) Pentium (R) CPU N3540 @ 2.16 GHz (4CPUs)
RAM	8192 MB

**Table 4:** Parameters used to generate 10,000 scenarios for inventory policies obtained by SP in two stages.

Statistical Parameters	Inventory Parameters
•μ∼U(300,1200)	$•\;h_{t}∼U\left(0.25,0.33\right)$
•σ∼U(10,60)	$•\;o_{t}∼U\left(12,000,18,000\right)$
	$•\;u_{t}∼U\left(15,000,30,000\right)$
	$•\;s_{t}∼U\left(700,2000\right)$
	$•\;C_{t}∼U\left(10,000,000,20,000,000\right)$

**Table 5:** Descriptive statistics for the indicators of the 10,000 scenarios of the simulation study.

Statistic	μ	σ	SD[Y]	CS[Y]	CK[Y]	*Q*	*I*	*S*	TC	*o*	*h*	*s*	*u*	*C*
Minimum	300.10	10.01	688.00	−1.73	2.93	280.50	3.11	2.50	4,791,629.00	12,003.00	0.25	700.20	15,133.00	100,007,582.00
1st quartile	454.30	22.47	1764.00	−1.04	3.96	441.40	10.11	9.90	40,749,989.00	13,481.00	0.27	1024.30	64,455.00	129,817,770.00
Median	633.30	35.09	2468.00	−0.93	4.38	617.40	15.76	15.67	72,711,355.00	14,980.00	0.29	1358.90	114,840.00	156,133,623.00
Mean	671.90	35.11	2698.00	−0.93	4.52	653.30	19.56	19.93	75,374,976.00	14,989.00	0.29	1357.90	125,808.00	154,022,583.00
3rd quartile	869.00	47.85	3437.00	−0.82	4.90	844.30	24.36	25.27	104,361,290.00	16,466.00	0.31	1692.00	176,845.00	178,923,427.00
Maximum	1199.70	60.00	6362.00	−0.39	17.45	1191.20	110.94	120.44	196,036,684.00	17,998.00	0.33	2000.00	299,964.00	199,984,413.00

**Table 6:** Pearson correlation coefficient matrix between variables of the simulation study.

	*C*	*h*	*I*	CK[Y]	μ	*o*	*Q*	*s*	*S*	SD[Y]	CS[Y]	σ	TC	*u*
*C*	1.00	−0.02	0.03	−0.00	0.06	0.02	0.06	−0.01	0.03	0.05	−0.01	0.01	0.22	0.14
*h*	−0.02	1.00	−0.00	0.00	−0.01	−0.00	−0.01	0.02	−0.01	−0.01	0.01	0.01	−0.01	−0.01
*I*	0.03	−0.00	1.00	−0.31	0.55	−0.01	0.54	0.00	0.73	0.05	0.43	−0.67	0.12	−0.22
CK[Y]	−0.00	0.00	−0.31	1.00	−0.02	−0.01	−0.01	0.01	−0.29	0.19	−0.25	0.39	−0.00	0.01
μ	0.06	−0.01	0.55	−0.02	1.00	−0.01	1.00	0.01	0.55	0.83	0.03	−0.04	0.23	−0.38
*o*	0.02	−0.00	−0.01	−0.01	−0.01	1.00	−0.01	0.00	−0.02	−0.01	−0.01	−0.00	0.01	0.01
*Q*	0.06	−0.01	0.54	−0.01	1.00	−0.01	1.00	0.01	0.51	0.84	0.01	−0.01	0.24	−0.38
*s*	−0.01	0.02	0.00	0.01	0.01	0.00	0.01	1.00	−0.00	0.01	−0.00	0.00	−0.01	−0.01
*S*	0.03	−0.01	0.73	−0.29	0.55	−0.02	0.51	−0.00	1.00	0.06	0.43	−0.64	0.11	−0.20
SD[Y]	0.05	−0.01	0.05	0.19	0.83	−0.01	0.84	0.01	0.06	1.00	−0.27	0.49	0.21	−0.32
CS[Y]	−0.01	0.01	0.43	−0.25	0.03	−0.01	0.01	−0.00	0.43	−0.27	1.00	−0.55	−0.00	−0.01
σ	0.01	0.01	−0.67	0.39	−0.04	−0.00	−0.01	0.00	−0.64	0.49	−0.55	1.00	0.01	0.01
TC	0.22	−0.01	0.12	−0.00	0.23	0.01	0.24	−0.01	0.11	0.21	−0.00	0.01	1.00	0.75
*u*	0.14	−0.01	−0.22	0.01	−0.38	0.01	−0.38	−0.01	−0.20	−0.32	−0.01	0.01	0.75	1.00

**Table 7:** Extract of correlations of interest for the simulation study according to their significance given by its *p*-value.

Variables	Type of Correlation	*p*-Value
*I* versus CK[Y]	Inverse	<0.0001
*I* versus μ	Direct	<0.0001
*I* versus *S*	Direct	<0.0001
*I* versus CS[Y]	Direct	<0.0001
*S* versus CK[Y]	Inverse	<0.0001
*S* versus μ	Direct	<0.0001
*S* versus CS[Y]	Direct	<0.0001

**Table 8:** Results of linear regression between variables with significant correlations for the simulation study.

Response Variable	Covariate	Intercept	Slope	Adjusted-$R^{2}$
*I*	CK[Y]	427.35	−51.29	0.10
*I*	μ	−6,338,260.00	0.03	0.31
*I*	CS[Y]	527.79	355.66	0.19
*S*	CK[Y]	432.58	−51.65	0.09
*S*	μ	−13,702.13	0.03	0.30
*S*	CS[Y]	547.00	372.33	0.18

**Table 9:** Mean of the groups of variables for the different scenarios of the simulation study.

Group	*I*	CK[Y]	μ	*Q*	*S*	SD[Y]	CS[Y]	TC
1	19.14	4.53	648.93	631.16	19.24	2597.06	−0.93	66,597,107.00
2	18.18	4.50	621.31	604.12	18.52	2493.29	−0.93	28,205,097.00
3	19.79	4.52	695.12	675.42	20.76	2796.15	−0.92	103,328,543.00
4	23.25	4.49	794.72	773.61	23.06	3209.21	−0.93	147,193,441.00

**Table 10:** Results of the fit with a multinominal logistic regression model for the simulation study.

Group	Intercept	CK[Y] (Slope)	CS[Y] (Slope)
2	0.18	−0.04	0.01
3	0.12	−0.01	0.25
4	−0.72	−0.06	−0.15

**Table 11:** Probability of belonging to the groups according to the CS and CK for the simulation study.

Group	Coefficient	Value of the Coefficient	Probability (Group)
	CK[Y]	2.92 (min)	0.49
2		17.45 (max)	0.63
	CS[Y]	−1.72 (min)	0.46
		−0.38 (max)	0.46
	CK[Y]	2.92 (min)	0.71
4		17.45 (max)	0.85
	CS[Y]	−1.72 (min)	0.62
		−0.38 (max)	0.66

**Table 12:** Descriptive statistics for the monthly drug DPUT data.

Statistic	Value
Minimum (units/month)	84.01
1st quartile (units/month)	106.34
Median (units/month)	113.39
Mean (units/month)	112.29
3rd quartile (units/month)	121.29
Maximum (units/month)	129.95
CS (dimensionless)	−0.64
CK (dimensionless)	3.75

**Table 13:** Estimated parameters of the WEI3 distribution with monthly drug DPUT data.

Estimated Parameters	Value
μ	112.00
σ	12.30
SD[Y]	267.56

**Table 14:** Coefficients to carry out the two-stage SP in case study with monthly drug DPUT data.

$u_{t}$	$h_{t}$	$o_{t}$	$s_{t}$	$C_{t}$	TC	$Z_{t}$	$Q_{t}$	$S_{t}$	$I_{t}$
($/Unit)	($/Unit)	($/Order)	($/Unit)	($)	($)	(Binary Unit)	(Units)	(Units)	(Units)
120,000.00	0.29	16,000.00	1200.00	2.50 $\times \;10^{7}$	13,127,477.00	1.00	109.00	5.79	7.27

**Table 15:** Evaluation of out-of-sample scenarios for the case study according to the listed indicator.

Performance Indicator	GAMLSS Framework	Robust Counterpart
E(Δ)	2.50	−41.50
MAD	2.50	41.50

## Data Availability

The data analyzed are available under request.
